# HMDD v3.0: a database for experimentally supported human microRNA–disease associations

**DOI:** 10.1093/nar/gky1010

**Published:** 2018-10-26

**Authors:** Zhou Huang, Jiangcheng Shi, Yuanxu Gao, Chunmei Cui, Shan Zhang, Jianwei Li, Yuan Zhou, Qinghua Cui

**Affiliations:** 1 Department of Biomedical Informatics, Department of Physiology and Pathophysiology, Center for Noncoding RNA Medicine, MOE Key Lab of Cardiovascular Sciences, School of Basic Medical Sciences, Peking University, 38 Xueyuan Rd, Beijing 100191, China; 2 Institute of Computational Medicine, School of Artificial Intelligence, Hebei University of Technology, Tianjin 300401, China; 3 Center of Bioinformatics, Key Laboratory for Neuro-Information of Ministry of Education, School of Life Science and Technology, University of Electronic Science and Technology of China, Chengdu 610054, China

## Abstract

Comprehensive databases of microRNA–disease associations are continuously demanded in biomedical researches. The recently launched version 3.0 of Human MicroRNA Disease Database (HMDD v3.0) manually collects a significant number of miRNA–disease association entries from literature. Comparing to HMDD v2.0, this new version contains 2-fold more entries. Besides, the associations have been more accurately classified based on literature-derived evidence code, which results in six generalized categories (genetics, epigenetics, target, circulation, tissue and other) covering 20 types of detailed evidence code. Furthermore, we added new functionalities like network visualization on the web interface. To exemplify the utility of the database, we compared the disease spectrum width of miRNAs (DSW) and the miRNA spectrum width of human diseases (MSW) between version 3.0 and 2.0 of HMDD. HMDD is freely accessible at http://www.cuilab.cn/hmdd. With accumulating evidence of miRNA–disease associations, HMDD database will keep on growing in the future.

## INTRODUCTION

MicroRNAs (miRNAs) are an important class of small non-coding RNA molecules that regulate gene expression by targeting mRNAs for cleavage or translational repression (1,2). With the development of high-throughput experimental techniques, researchers have confirmed ∼2600 miRNAs, which are likely to target more than 60% of human protein-coding genes (3,4).

**Figure 1.**
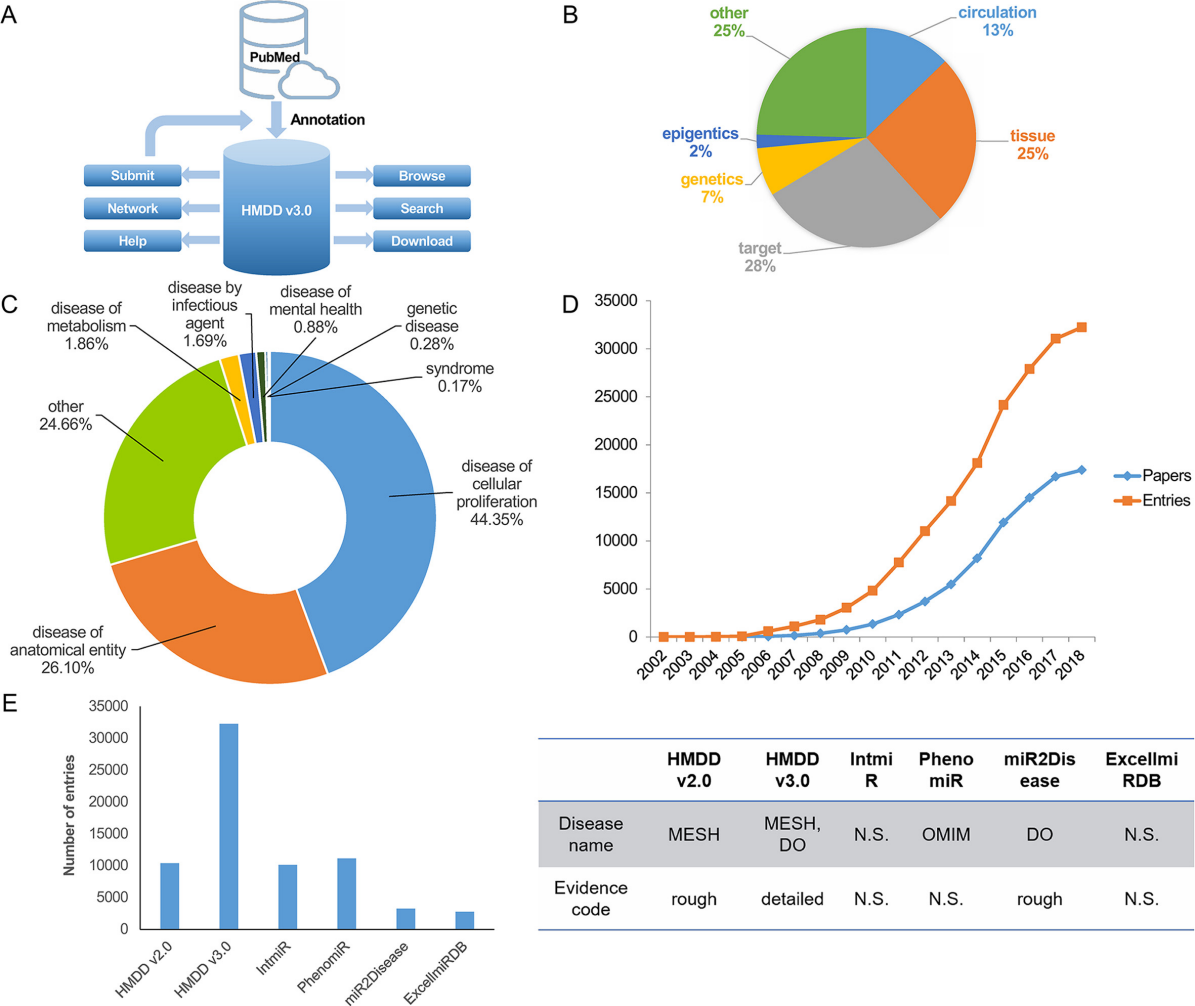
Overview of the HMDD v3.0 database. (**A**) The schematic overview of HMDD v3.0. (**B**) Pie chart depicting the fractions of entries from six evidence categories. (**C**) Circular chart showing the distribution of all 850 diseases on the basis of disease classification from Disease Ontology. (**D**) Cumulative counts of miRNA–disease entries per publication time. (**E**) The comparison between HMDD v3.0 and other miRNA–disease association databases. N.S., non-specific or non-standardized.

Given the important functionality of miRNAs, dysregulation of miRNAs is associated with a large number of diseases, such as cancer, cardiovascular diseases, and neurodegenerative diseases (5). Therefore, a database for miRNA–disease association is important for biomedical scientists investigating the roles of miRNAs in diseases, and for bioinformatics scientists discovering patterns of miRNAs in diseases and developing novel miRNA–disease association prediction algorithms. For this purpose, we built the first version of human microRNA disease database (HMDD) on December 2007 (6) and released the second version on June 2013 (7). During the past 11 years, we have updated it more than 30 times to ensure that HMDD could keep up with research advances in recent years. However, the roles of miRNAs in diseases are prominently diverged. For example, miRNAs can both promote and suppress cancers occurrence and progression, and they can serve as diagnosis and prognosis biomarkers (8–11) and novel therapeutic targets for the treatment of cancers (12,13). Therefore, it is of urgent demand to update the database for more comprehensive data coverage and more accurate classifications of the miRNA–disease association evidence. To this end, we adopted an improved pipeline for manual data curation (Figure 1A) and launched v3.0 version of HMDD. Currently, HMDD v3.0 has collected 32281 experimentally supported miRNA–disease association entries, covering 1102 miRNA genes, 850 diseases from 17412 papers. As the result, there is about two-fold increment of data, comparing to HMDD v2.0, which collected 10368 entries that include 572 miRNA genes, 378 diseases from 3511 papers. According to experimental evidence, we classify these entries into six categories covering 20 different evidence codes (Table 1). A more detailed description of the updated database is available at the following sections.

**Table 1.** The six categories of evidence covering 20 different evidence codes (‘ns’ means ‘non-specific’)

Class	Code	The number of entries
Circulation	circulation_biomarker_diagnosis_up	994
Circulation	circulation_biomarker_prognosis_up	115
Circulation	circulation_biomarker_diagnosis_down	559
Circulation	circulation_biomarker_prognosis_down	64
Circulation	circulation_biomarker_diagnosis_ns	2110
Circulation	circulation_biomarker_prognosis_ns	278
Tissue	tissue_expression_down	2442
Tissue	tissue_expression_up	2835
Tissue	tissue_expression_ns	2953
Genetics	genetics_knock down_promote	225
Genetics	genetics_knock down_suppress	199
Genetics	genetics_overexpression_promote	275
Genetics	genetics_overexpression_suppress	568
Genetics	genetics_GWAS	1035
Epigenetics	epigenetics	644
Target	therapeutic target	2080
Target	lncRNA target	267
Target	target gene	6356
Target	transcription factor target	361
Other	other	7921

## DATA COLLECTION AND OVERVIEW

The main purpose of HMDD v3.0 is to provide a convenient and comprehensive web-based resource where users can search, browse, download and analyze the experimentally supported miRNA–disease associations. To compile the dataset, similar to HMDD v2.0 (7), we used the keywords ‘microRNA’, ‘miRNA’ or ‘miR’ to obtain microRNA-related papers from PubMed and downloaded these publications’ abstracts. After that, we read these abstracts to extract information about miRNA–disease associations, which contains miRNA name, disease name, the publication PubMed ID (PMID) and evidence supporting the relationship between miRNA and disease. Then, we performed classification of miRNA–disease associations according to the evidence. Figure 1B depicts the overall view of the six categories of evidence, where the ‘target’ and ‘tissue’ categories composites the largest fraction of experimental reports. Besides, we also re-curated the miRNA–disease association data from TAM 2.0 server (14) if they were not included. For each miRNA–disease association entry, we also standardized miRNA names on the basis of the miRBase (http://www.mirbase.org/) (3) and the disease names mainly according to Disease Ontology (http://www.disease-ontology.org) (15) and MeSH Terms (https://www.ncbi.nlm.nih.gov/mesh). According to the disease hierarchy from Disease Ontology (15), we further grouped diseases into eight types (Figure 1C).

The cumulative count of miRNA–disease entries per year is summarized in Figure 1D. Since the publication of HMDD v2.0, the accumulation of experimental reports has kept on accelerating, which again signifies the necessity of HMDD updates. Interestingly, the studies that focus on the expression of miRNA in circulation system or lesion tissues contribute the most to the growth of data, which indicates the wide application of transcriptome profiling for screening potential miRNA biomarkers and therapeutic targets. However, such kind of experiential evidence is often weak or preliminary. In HMDD v3.0, we provided detailed evidence code for each miRNA–disease entry, so that the users could assess the confidence level of the miRNA–disease associations. In all, the detailed evidence code classification and disease name standardization, together with the significant data accumulation, constitute the major improvement of HMDD v3.0, as depicted in Figure 1E that compares the HMDD v3.0 with HMDD v2.0 and other human miRNA–disease association databases (7,16–19).

## DATABASE USAGE

The HMDD v3.0 website is now available at http://www.cuilab.cn/hmdd, by using the SQLite+Django framework. Users can browse all the hierarchical structure of the database in the ‘Browse’ page. The entries are firstly grouped based on their evidence categories, which constitute the top node in the hierarchical tree. Under one category, user can choose a miRNA or a disease to obtain the related entries. Each entry is supplemented with its reference PMID and description of the experimental evidence, and details of miRNA or disease terms are accessible by clicking the links on the miRNA/disease name. For each miRNA, links to miRBase (3) and the functional enrichment information of its target genes (14) are provided. Briefly, we obtained the known functional gene sets (including Hallmarks, GO, KEGG) from MSigDB (20), and target genes from miRWalk. Since the target data of miRWalk (21) include predicted results and experimental evidence, the target gene set for each miRNA was defined as the consensus of both two kind of target data. Then we performed gene set enrichment analysis for each target gene set using hypergeometric test. Finally, the *P* values for all signature gene sets are adjusted by Benjamini-Hochberg correction. As the miRNAs of HMDD are organized at the miRNA precursor level, the enriched functions of each mature miRNA are aggregated to the corresponding miRNA precursor. As for the disease terms, they are linked to various disease nomenclature databases including ICD10CM (https://www.icd10data.com/), MESH (https://www.ncbi.nlm.nih.gov/mesh), OMIM (http://omim.org/), DOID (http://www.disease-ontology.org/) and HPO (https://hpo.jax.org/app/).

Alternatively, users can utilize the ‘Search’ function in the website. They can quickly obtain the results by simply entering the full miRNA/disease name (exact mode) or the miRNA/disease keyword (fuzzy mode) in the searching field. The batch searching is also allowed if multiple (at maximum 20) semicolon-delimited keywords are entered. The searching results can be downloaded by clicking the button above the result table. Thirdly, users could not only download all the datasets in the ‘Download’ page and but also submit their own evidence in the ‘Submit’ page. For non-specialists, we described the HMDD usage in more details and offer them guidelines in the ‘Help’ page of website. Finally, we added a function for the network visualization of (disease-context) miRNA–target interaction, based on the experimentally supported miRNA–target data from miRTarBase (22). Users can click one disease to view the interaction network between the miRNAs and genes associated with this disease (the disease genes are from DisGeNET (23)). Besides, we added the regulation pattern (up/down) between miRNA and genes, using the annotations from TarBase v8 (24). Alternatively, if users click one miRNA name, all disease genes targeted by this miRNA will be illustrated in the similar network fashion.

## EXAMPLE OF DATA UTILITY: ANALYSIS OF DSW AND MSW

**Figure 2.**
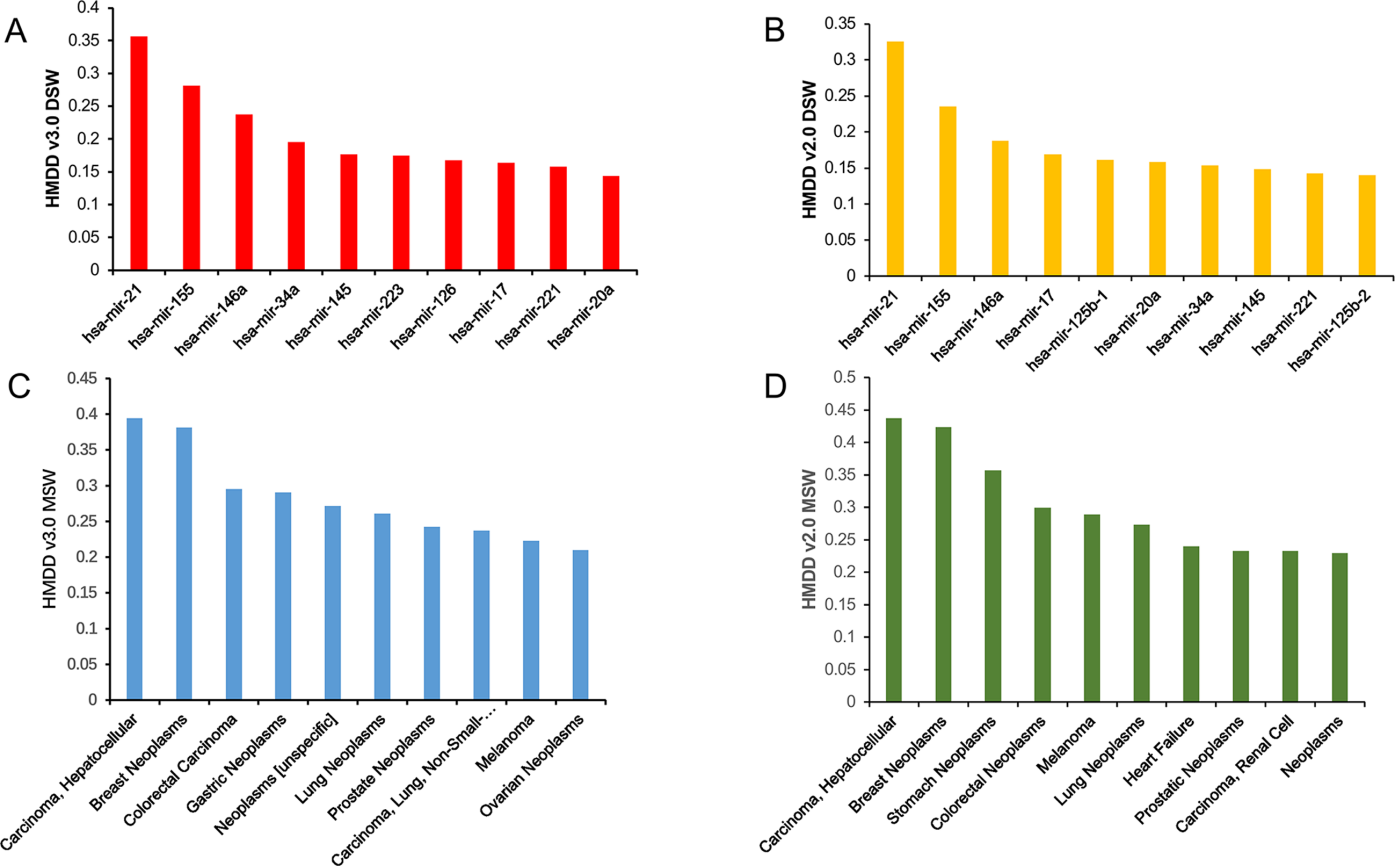
Comparison of DSW and MSW scores between HMDD v3.0 and HMDD v2.0. (**A**) The top 10 miRNAs with the highest DSWs in HMDD v3.0. (**B**) The top 10 miRNAs with the highest DSWs in HMDD v2.0. (**C**) The top 10 miRNAs with the highest MSWs in HMDD v3.0. (**D**) The top 10 miRNAs with the highest MSWs in HMDD v2.0.

The importance of one miRNA in human diseases can be roughly assessed by its disease spectrum width (DSW), a straightforward measurement that was proposed by us in a previous research (25). The DSW of one miRNA *i* reads:

\begin{equation*}{\rm DSW}(i) = n(i){\rm{/}}N\end{equation*}

where *n*(*i*) is the number of diseases associated with miRNA *i, N* is the total number of diseases in the database (25). We compared the DSW scores based on HMDD v3.0 and HMDD v2.0 (Figure 2A and B). Interestingly, the top three miRNAs are shared, i.e. miR-21 (0.36), miR-155 (0.28) and miR-146a (0.24) comparing to HMDD v2.0 (miR-21: 0.33, miR-155: 0.24, miR-146a: 0.19), suggesting that they are associated with many diseases and have important roles in multiple biological processes. Besides, their DSWs are higher in the HMDD v3.0 results, indicating the novel disease association of these miRNAs have been continuously proposed in recent years. Finally, since the disease association spectrum of these miRNAs are especially wide, they are not likely to server as biomarkers since the disease specificity is not satisfactory.

In the HMDD v2.0 (7), we introduced a similar measurement for each disease, i.e. miRNA spectrum width (MSW) of a disease. If one disease is associated with many miRNAs, its underlying mechanism would involve a complicated miRNA regulatory network. Therefore, MSW could be used to preliminarily evaluate the complexity of a disease (7). The top 10 diseases with the highest MSWs based on HMDD v3.0 and HMDD v2.0 were show in Figure 2C and D. Clearly, cancers, as the well-acknowledged complex diseases, dominate the top list of diseases, though there are marginal differences in the detailed rank of diseases between HMDD v3.0 and HMDD v2.0. The analysis results of DSW and MSW are also provided in the ‘Download’ page of HMDD v3.0 website for further investigations.

## CONCLUSION

miRNAs are showing increasing importance in numerous diseases, and their potential ability of being diagnosis and prognosis biomarkers and therapeutic targets have been repeatedly demonstrated. Therefore, it is necessary to build and update miRNA–disease association databases to include the recent studies. HMDD v3.0 integrated many past publications about miRNA–disease associations, and offered evidence-stratified miRNA–disease data based on six categories of 20 evidence codes. HMDD v3.0 also provided two new function modules: (target gene) functional enrichment information of each miRNA and miRNA–target network online visualization. As a demonstration of database usefulness, MSW and DSW metrics were calculated and made available at the website. The important roles of several miRNAs have been confirmed by comparing the DSW scores derived from HMDD v3.0 and those derived from HMDD v2.0. Moreover, HMDD datasets could also be used for various analyses including but not limited to tracing the research hotspot, predicting miRNA-associated disease and performing functional enrichment analysis. Indeed, there were several successful prediction tools for miRNA–disease associations (26,27) based on the previous versions of HMDD, and therefore the HMDD v3.0 data could serve as the expanded training dataset for further improvement of such tools. Furthermore, besides HMDD, there are also some other miRNA–disease databases, such as miR2Disease (18), PhenomiR (28), dbDEMC (29), the data in HMDD could be used to rapidly update these databases by their authors. Finally, we believe HMDD v3.0 represents an important and useful resource for investigating miRNA–disease associations. With continuous updating of HMDD, users will get more comprehensive information about miRNA–disease associations in the future.
